# Citrullination of DNMT3A by PADI4 regulates its stability and controls DNA methylation

**DOI:** 10.1093/nar/gku522

**Published:** 2014-06-21

**Authors:** Rachel Deplus, Hélène Denis, Pascale Putmans, Emilie Calonne, Marie Fourrez, Kazuhiko Yamamoto, Akari Suzuki, François Fuks

**Affiliations:** 1 Laboratory of Cancer Epigenetics, Faculty of Medicine, Université Libre de Bruxelles, 808 route de Lennik, 1070 Brussels, Belgium; 2 Laboratory for Autoimmune diseases, Center for Integrative Medical Science, The Institute of Physical and Chemical Research (RIKEN), 1–7–22 Suehirocho, Tsurumi-ku, Yokohama, Kanagawa, Japan

## Abstract

DNA methylation is a central epigenetic modification in mammals, with essential roles in development and disease. *De novo* DNA methyltransferases establish DNA methylation patterns in specific regions within the genome by mechanisms that remain poorly understood. Here we show that protein citrullination by peptidylarginine deiminase 4 (PADI4) affects the function of the DNA methyltransferase DNMT3A. We found that DNMT3A and PADI4 interact, from overexpressed as well as untransfected cells, and associate with each other's enzymatic activity. Both *in vitro* and *in vivo*, PADI4 was shown to citrullinate DNMT3A. We identified a sequence upstream of the PWWP domain of DNMT3A as its primary region citrullinated by PADI4. Increasing the PADI4 level caused the DNMT3A protein level to increase as well, provided that the PADI4 was catalytically active, and RNAi targeting PADI4 caused reduced DNMT3A levels. Accordingly, pulse-chase experiments revealed stabilization of the DNMT3A protein by catalytically active PADI4. Citrullination and increased expression of native DNMT3A by PADI4 were confirmed in PADI4-knockout MEFs. Finally, we showed that PADI4 overexpression increases DNA methyltransferase activity in a catalytic-dependent manner and use bisulfite pyrosequencing to demonstrate that PADI4 knockdown causes significant reduction of CpG methylation at the p21 promoter, a known target of DNMT3A and PADI4. Protein citrullination by PADI4 thus emerges as a novel mechanism for controlling a *de novo* DNA methyltransferase. Our results shed new light on how post-translational modifications might contribute to shaping the genomic CpG methylation landscape.

## INTRODUCTION

DNA methylation is a key mechanism of epigenetic regulation in eukaryotes. In mammals, DNA methyltransferases (DNMTs) establish and maintain DNA, methylation of the fifth carbon of cytosine residues within CG dinucleotides (1). There are three enzymatically active mammalian DNMTs: DNMT1, DNMT3A and DNMT3B, and one related regulatory protein, DNMT3L, which lacks catalytic activity. DNMT1 is primarily a maintenance methyltransferase ensuring the inheritance of proper DNA methylation patterns in differentiated somatic cells (2). In mammals, *de novo* DNA methylation is mediated essentially by DNMT3A and DNMT3B, which knockout studies in mice have shown to be essential to embryonic development (3). DNA methylation plays a crucial role in maintaining cell pluripotency, X-chromosome inactivation and genomic imprinting (4), while aberrant DNA methylation is the best-characterized epigenetic hallmark of several pathologies, including cancers (5). Dysregulated expression of DNMTs has also been reported in various human cancers (6).

DNA methylation is not randomly distributed in the mammalian genome, and the mechanisms underlying the generation of CpG methylation patterns are still poorly understood. Accruing evidence suggests several mechanisms for the preferential targeting of DNMTs to certain genomic loci (7). First, chromatin modifications, and particularly histone modifications, can influence the DNA methylation pattern by guiding DNMTs to specific genomic regions (7). For instance, genome-wide analysis has shown that DNMT3A is excluded from active chromatin marked by H3K4 trimethylation (8). Other histone modifications, including H3K36me3, H3K9me3 and H3K27me3 (7) have also been implicated in guiding DNA methylation to specific chromatin regions. Second, DNMTs can be recruited to specific sequences by associating with certain transcription factors. For instance, DNMTs recruited by the oncogenic transcription factors PML-RAR (9) and Myc (10) have been shown to methylate and silence specific target promoters. A third emerging mechanism is the regulation of DNMTs by post-translational modifications (PTMs). Biochemical studies have shown that this regulation occurs (7), but the endogenous modifications and enzymes involved remain to be discovered. Acetylation, SUMOylation, methylation and phosphorylation are all reported to influence the function of DNMT1 as regards its catalytic properties, stability and interaction with other proteins (7,11,12). The only PTM reported to occur in *de novo* DNMTs is SUMOylation, observed *in vitro* with DNMT3A and DNMT3B (13,14).

Peptidylarginine deiminases (PADs) are Ca^++^-dependent enzymes catalyzing the conversion of arginine residues to citrulline in a PTM process called citrullination (also referred to as deimination or demethylimination). PADI4 is the PAD isoform located in the nucleus, known to citrullinate both histones (H2A, H3 and H4) (15–17) and non-histone proteins. Citrullination of the substrate benzoyl-L-arginine ethylester by PADI4 requires its activation through binding of calcium ions. Essential to PADI4 activity are the residues Asp350, His471, Asp473 and Cys645, located in the active site cleft (18). Functionally, PADI4 catalyzes histone citrullination at the estrogen-regulated pS2 promoter (15,19,20) and at the apoptosis-related gene promoters *p21* and *OKL38* (21,22), thereby repressing gene transcription. How PADI4 silences gene expression is not yet known, but we and others have proposed that PADI4 and histone deacetylases collaborate to generate a repressive chromatin environment (19,23). Interaction of PADI4 with HDAC1 and HDAC2 leads to recruitment of these proteins and the corresponding activities to the pS2 and p21 promoters, where they may act as co-repressors of gene expression (19,23). PADI4 might also function as a transcriptional co-activator of at least a subset of genes. In 2011, Zhang *et al.* performed a global analysis showing enrichment of the PADI4-bound DNA fraction in certain gene promoters of actively transcribed regions (24). More recently, citrullination by PADI4 was shown to weaken the binding of HP1 to trimethylated histone H3 lysine 9, causing transcriptionnal derepression (25). Although histones are the best-studied PADI4 substrates, there is now increasing evidence that certain non-histone proteins are also citrullinated by PADI4. These include the chaperone protein nucleophosmin/B23 (26,27), the transcriptional coactivators p300 and Elk-1 (24,28) and the tumor suppressor protein ING4 (29).

As little is known about PTMs of *de novo* DNMTs, we have examined here whether these enzymes might be targets of PADI4-catalyzed citrullination. We report that PADI4 interacts with, stabilizes and citrullinates DNMT3A both *in vitro* and *in vivo*. We show that a region just upstream from the PWWP domain of DNMT3A is the primary citrullinated region. We demonstrate citrullination and increased expression of DNMT3A by PADI4 in mouse embryonic fibroblasts (MEFs) derived from PADI4-deficient mice. Lastly, we find that these observations correlate with a PADI4-catalysis-dependent impact of PADI4 on CpG methylation of D4Z4 repeats and of the p21 promoter. These data suggest that PADI4 may play a role in DNA methylation by stabilizing and citrullinating DNMT3A. Taken together, they identify PADI4-mediated citrullination as a new mode of *de novo* DNMT activity regulation. This work sheds new light on the origin of DNA methylation patterns and on the role played by PTMs in controlling DNMT function.

## MATERIALS AND METHODS

### Expression plasmids and site-directed mutagenesis

We cloned the sequences encoding full-length PADI4 and mutant PADI4 (C645S) into the pLPC retroviral vector. The C645S PADI4 mutant was produced with the QuickChange Site-directed Mutagenesis kit (Stratagene) according to the manufacturer's instructions. The following plasmids have been described previously: pGEX PADI4 full-length (30), pcDNA3.1-HA PADI4 full-length (19), pRS PADI4 (19), pcDNA3.1-GAL4 DNMT3A full-length (10), pcDNA3.1-myc DNMT3A full-length (31), pET-30 DNMT3A full-length (10) and pET-30 H3 (32). We cloned the sequences encoding various DNMT3A fragments and histone H3 full length into the pET30a vector by polymerase chain reaction (PCR) using appropriate sets of primers.

### GST fusion and His-tagged proteins, *in vitro* translation and pull-down assays

Recombinant GST-fused and His-tagged proteins were expressed in and purified from *Escherichia coli* BL21 as described (33). We used the TNT system (Promega) to carry out *in vitro* transcription/translation. DNMT3A gene was *in vitro* transcribed/translated from pcDNA3.1-myc DNMT3A. GST pull-down experiments were performed as described previously (33).

### Cell culture and transient transfections

293T, 293GP, U2OS and HCT116 DKO cells were maintained in Dulbecco's modified Eagle's medium (DMEM; Invitrogen) supplemented with 10% fetal calf serum. Transient transfections were performed with polyethylene imine (Euromedex) as described previously (34). We used E14.5 mice knockout for PADI4 (35) for making MEFs. Culture medium is DMEM+10% FBS (Fetal Bovine Serum) +1% penicillin and 1% streptomycin. After trypsinization, a cell strainer was used for obtaining single-cell suspension and then cell suspension was cultured using culture medium in a dish (10 cm dish/1 embryo). Cells were cultured at 37°C in 5% CO_2_ atmosphere until confluent. These MEFs were maintained for a maximum of 5 passages. To obtain cells synchronized during different phases of the cell cycle, U2OS cells were incubated with nocodazole (20 ng/ml) for 16 h with or without a released of 45 min in fresh medium. U2OS cells were treated with thymidine (2 mM) for 16 h, washed, released for 8 h into fresh medium and treated again with thymidine for 16 h. After the double-thymidine block, cells were washed twice with phosphate buffered saline (PBS), collected or released in complete medium for 3 h or 8h to obtain cells in G1/S or S phases, respectively.

### Cell extracts, immunoprecipitations and western blot analyses

Whole-cell extracts were prepared in IPH lysis buffer (50 mM Tris-HCl pH 8, 150 mM NaCl, 5mM ethylenediaminetetraacetic acid (EDTA), 0.5% NP40). Nuclear extracts were prepared as follows: cells were first lysed in buffer A (10 mM HEPES pH 7.9, 10 mM KCl, 0.1 mM EDTA, 1 mM DTT, protease inhibitors) and centrifuged at 10 000 *g* for 5 min. Buffer B (20 mM HEPES pH7.9, 0.4 M NaCl, 1 mM EDTA, 10% glycerol, 1 mM DTT, protease inhibitors) was then added to the nuclear pellet fraction, which was shaken vigorously for 2 h and centrifuged at 10 000 *g* for 10 min. The supernatant (nuclear fraction) was aliquoted and stored at –80°C until used. All procedures were performed at 4°C. For DNAse treatments, nuclear extracts were incubated with an excess of DNAse I for 1 h at 37°C prior to immunoprecipitation. The effectiveness of DNAse treatment was checked on plasmidic DNA (see Supplementary Figure S1).

Standard procedures were used for immunoprecipitation and western blotting (10). The primary antibodies used in these experiments were directed against the following: control immunoglobulin G (IgG) (sc-2027; Santa Cruz), HA (ab18181; Abcam), GAL4 (sc- 510; Santa Cruz), DNMT3A (sc-20703; Santa Cruz), PADI4 (ab18181; Abcam), Citrulline (ab100932; Abcam), Citrulline H3 (ab1791, Abcam), HDAC1 (pAb-053–050; Diagenode), Actin (Sigma; A5316) and TBP (ab818, Abcam).

### DNMT assay

The DNMT assay of Active Motif was used. This method measures methylation of a CpG-rich DNA substrate by means of a sensitive enzyme-linked immunosorbent assay exploiting the high-affinity binding of methyl CpG binding domain (MBD) proteins methylated DNA. In some experiments, GST-PADI4 was incubated with HeLa nuclear extract (Computer Cell Culture Center, Belgium) (the source of DNMTs), pulled-down, washed and then assayed for associated DNMT activity. In others, nuclear extracts prepared from stably transfected cells and containing equal amounts of nuclear proteins were assayed directly.

### Histone citrullination assay

The assay was done essentially as described previously (15,19). Whole-cell extracts of transfected 293T cells were incubated overnight at 4°C with the relevant antibodies. Antibody complexes were collected on protein A/G-Sepharose beads, washed and then tested for histone deiminase activity. The reaction mixture containing 100 mM Tris-HCl (pH 7.6), 5 mM DTT, 10 mM CaCl_2_ and 10 μg histones (Roche) was incubated at 37°C for 1 h. Reaction products were then resolved by sodium dodecylsulphate-polyacrylamide gel electrophoresis (SDS-PAGE) and western blotted with anti-histone H3 (citrulline 17+2+8) antibody (ab5103; Abcam).

### Protein citrullination assay

Whole-cell extracts of transfected U2OS cells were incubated overnight at 4°C with the relevant antibodies. Antibody complexes were then collected on protein A/G-Sepharose beads, washed and tested for deiminase activity. The reaction mixture containing 100 mM Tris-HCl (pH 7.6), 5 mM DTT, 10 mM CaCl_2_ and recombinant His-DNMT3A was incubated at 37°C for 1 h. Reaction products were then resolved by SDS-PAGE and citrulline-containing proteins were detected with the Anti-Citrulline (Modified) Detection Kit (Millipore) according to the manufacturer's instructions.

### Protein stability assay

Protein stability was determined in a pulse-chase assay. 293T cells were transfected transiently with the indicated expression plasmid(s). Twenty-four hours post-transfection, the cells were washed with PBS, labeled for 15 min in methionine/cystein-free DMEM with [^35^S] methionine and [^35^S] cysteine (75 μCi/ml, TRAN^35^S-LABEL, MPBio), washed again with PBS and finally chased with complete DMEM. Cells were harvested at the indicated time points and total lysates were immunoprecipitated with anti-GAL4 antibodies. Proteins were subjected to SDS-PAGE, after which the gels were dried and autoradiographed at -80°C (supplier of the film: Amersham).

### RNA interference and retroviral infection

Briefly, 293 GP cells were transfected with the pRS and pLPC retroviral vectors. Supernatants were then collected and used to infect U2OS or HCT 116 DKO target cells.

### RNA purification and quantitative real-time PCR assays

Total RNA was extracted with the TriPure reagent (Roche) according to the manufacturer's instructions. DNase I treatment was performed with the DNA-free DNase kit (Ambion) according to the manufacturer's protocol. Quantitative PCRs were performed with SYBR Green dye (Eurogentec) on a LightCycler 480 (Roche). Briefly, Random hexamers (Invitrogen) and Superscript II reverse transcriptase (Invitrogen) were used to reverse-transcribe mRNAs to cDNAs. Gene expression levels were then evaluated by real-time PCR. GAPDH (Glyceraldehyde 3-phosphate dehydrogenase) was amplified as an internal control. The Primers used are listed in Supplementary Table S1.

### DNA extraction and bisulfite pyrosequencing

The methylation status of the D4Z4 repeat and p21 promoter was assessed by genomic bisulfite pyrosequencing. Genomic DNA was extracted with the QIAamp DNA Mini Kit (Qiagen), including the recommended proteinase K and RNase A digestions. One microgram of genomic DNA was then bisulfite-converted with the EpiTect Bisulfite Kit (Qiagen). Approximately half of the converted DNA was used as template in each subsequent PCR. Primers for PCR amplification and sequencing were deduced with the PyroMark® Assay Design 2.0 software (Qiagen). PCRs were performed with the HotStarTaq DNA polymerase PCR kit (Qiagen) under the following conditions: 95°C 15 min; 50 cycles of 95°C 30 s; 50°C 1 min; 72°C for 1min; 72°C for 10 min. The success of amplification was assessed by agarose gel electrophoresis, the PCR products were pyrosequenced with the Pyromark™ Q24 system (Qiagen). All primer sequences are listed in Supplementary Table S1.

### Statistics

Mann–Whitney tests were used to perform pairwise comparisons. All tests were performed with SPSS (PASW Statistics version 18.0).

## RESULTS

### DNMT3A associates with PADI4 *in vitro* and *in vivo*

**Figure 1.**
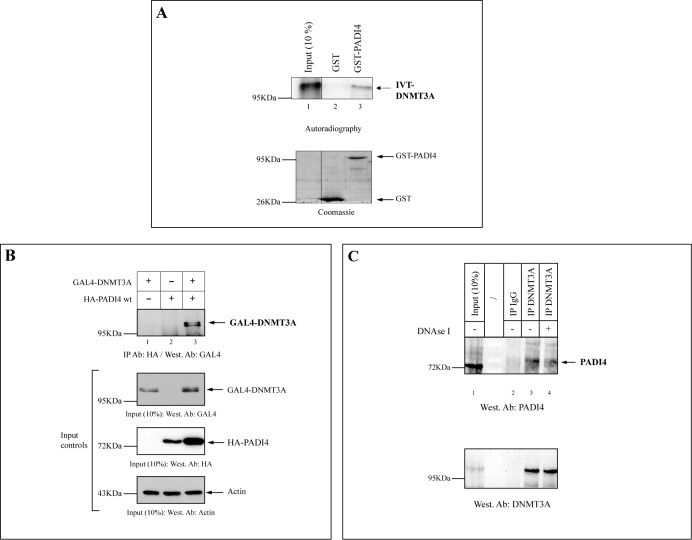
DNMT3A interacts with the histone deiminase PADI4. (A) DNMT3A binds to PADI4 *in vitro*. Full-length PADI4 fused to GST was tested in GST pull-down experiments in the presence of IVT full-length DNMT3A. Lane 1, input (10%) radiolabeled IVT-DNMT3A. (B) DNMT3A co-immunoprecipitates with PADI4 from transfected cells. 293T cells were transiently transfected with the indicated expression vector(s). Cell extracts were precipitated with anti-HA antibody and GAL4-DNMT3A was detected in the immunoprecipitates by western blot analysis. Input controls are shown (anti-GAL4 for DNMT3A, anti-HA for PADI4 and anti-Actin). (Of note, overexpression of DNMT3A appears to increase PADI4 expression, suggesting a positive feedback loop between PADI4 and DNMT3A.) (C) PADI4 co-immunoprecipitates with endogenous DNMT3A from untransfected U2OS cells. Cell extracts were immunoprecipitated with anti-DNMT3A or rabbit IgG and probed with antibodies against PADI4. 10% of the input loading control was used. Nuclear extracts were incubated or not with DNAse prior to immunoprecopitation (see Supplementary Figure S1 for effectiveness of DNAse treatment).

To learn more about how PTMs affect DNMT function, we focused on the *de novo* DNA methyltransferase DNMT3A, about which very little is known. We first performed pull-down assays to see if this enzyme might associate with the peptidylargininedeiminase PADI4, which catalyzes conversion of protein arginine residues to citrulline. As shown in Figure 1A, GST-fused full-length PADI4 was able to bind *in vitro* translated (IVT) radio-labeled full-length DNMT3A (Figure 1A, lane 3). GST failed to do so (lane 2). The gel was Coomassie blue stained to check for equal loading (Figure 1A, lower part). To further substantiate the interaction between DNMT3A and PADI4, we used co-immunoprecipitation assays. (It is worth noting that we used 293T and U2OS cells for most subsequent cell studies, consistently with several previous works on PADI4 (e.g. 23,35,36). In particular, 293T cells have often been employed for PADI4 overexpression studies, while U2OS cells have also been used for PADI4 overexpression and in endogenous/RNAi experiments.) As shown in Figure 1B, when 293T cells were transfected with GAL4-tagged DNMT3A and HA-tagged PADI4, we found DNMT3A to interact with PADI4 (Figure 1B, lane 3). No signal was detected in the precipitate after transfection of cells with either the GAL4-DNMT3A-encoding or HA-PADI4-encoding plasmid alone (Figure 1B, lanes 1 and 2, respectively). It is noteworthy that the level of DNMT3A protein in the total lysate was higher when both PADI4 and DNMT3A were ectopically expressed, suggesting a potential role for PADI4 in regulating DNMT3A protein stability (Figure 1B, Input controls/GAL4-DNMT3A, lane 3) (see below). To demonstrate an interaction between the endogenous PADI4 and DNMT3A proteins, we immunoprecipitated DNMT3A from untransfected U2OS cells and found the immunoprecipitate to contain PADI4 (Figure 1C, lane 3). Control (IgG) antibodies gave only a background signal (Figure 1C, lane 2). Since DNMT3A and PADI4 are both known to interact with chromatin (19,37), we also treated the lysates with DNAse to rule out the possibility that these proteins might co-immunoprecipitate because of indirect interactions mediated solely by chromatin (Figure 1C, lane 4 and Supplementary Figure S1). We also performed endogenous CoIP between PADI4 and DNMT3A in U2OS cells as above (cf. Figure 1C), this time using synchronized cells. After treatment of cells with nocodazole or thymidine, we observed association of DNMT3A with PADI4 in different phases of the cell cycle (G1, G1/S, S and M) (Supplementary Figure S2). These observations suggest no preferential interaction of PADI4 with DNMT3A during a specific phase of the cell cycle.

**Figure 2.**
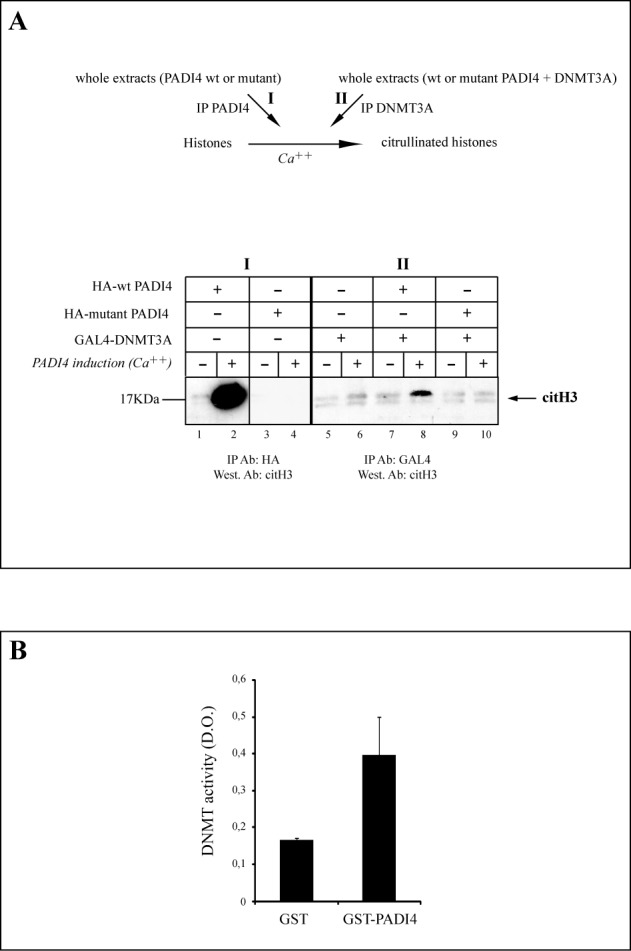
DNMT3A and PADI4 associate with each other's enzymatic activity. (A) 293T cells were transiently transfected with the expression vector(s) for GAL4-tagged DNMT3A and/or HA-tagged wt or catalytically defective PADI4 (18). Cell extracts were precipitated with anti-HA antibody (lanes 1–4) (IP PADI4) or anti-GAL4 antibody (IP DNMT3A) and the complexes were incubated with histones in presence and absence of calcium (Ca^++^). Histone deiminase activity was assayed by western blotting with anti-histone H3 (citrulline 17+2+8) (citH3). Vertical lines indicate juxtaposition of lanes non-adjacent within the same blot, exposed for the same time. (B) PADI4 associates with DNMT activity. Equivalent amounts of GST or GST-PADI4 fusion protein bound to Sepharose beads were incubated with HeLa nuclear extract, washed and assayed for associated DNMT activity.

Given the ability of PADI4 to citrullinate (deiminate) histones when presented with core histones (15,20), and in the light of the above data showing an interaction between DNMT3A and PADI4, we tested whether DNMT3A might associate with PADI4-mediated histone deiminase activity. As a preliminary experiment, we measured the deiminase activity of wild-type (wt) PADI4 and of a catalytically defective PADI4 mutant (C645S), using histones as substrates (Figure 2A). In a previous study where benzoyl-L-arginine ethylester was used as substate, the C645A mutant showed no detectable activity in the presence of Ca^2+^ (18). We transfected 293T cells with a vector expressing either wt or mutant HA-PADI4. Whole-cell extracts were prepared and precipitated with anti-HA antibody, and the immune complexes were added to purified core histones. A specific antibody was used to detect citrullinated histones H3 (citH3) (Figure 2A, scheme of the experiment (I)). In keeping with earlier reports (15,19), PADI4 proved able to deiminate histones, provided calcium ions were present (Figure 2A, lane 2 compared to lane 1). In contrast, the C645S mutant showed only background activity on histone substrates (Figure 2A, lane 4). The next step was to test the association of DNMT3A with PADI4 histone deiminase activity (Figure 2A, scheme of the experiment (II)). Upon GAL4-DNMT3A immunoprecipitation (Figure 2A, lanes 5–10), histone deiminase activity was found to co-purify with GAL4 only when the cells where co-transfected with the construct expressing wt PADI4 (Figure 2A, lane 8). No DNMT3A-associated PADI4 activity was observed after transfection with a vector expressing DNMT3A alone (Figure 2A, lane 6) or in cells co-expressing DNMT3A and the C645S PADI4 mutant (Figure 2A, lane 10).

Having shown that DNMT3A associates with PADI4-mediated histone citrullination, we examined whether PADI4 might associate with DNA methylation activity. This proved to be the case (Figure 2B): when incubated with HeLa nuclear extract, the pull-down obtained with GST-fused PADI4 was able to methylate a CpG-rich DNA substrate, whereas the pull-down obtained similarly with the GST control showed only background activity (Figure 2B). Taken together, our data thus indicate that PADI4 binds to DNMT3A and that in the complex formed, each enzyme remains active.

### PADI4 stabilizes the DNMT3A protein in mammalian cells

**Figure 3.**
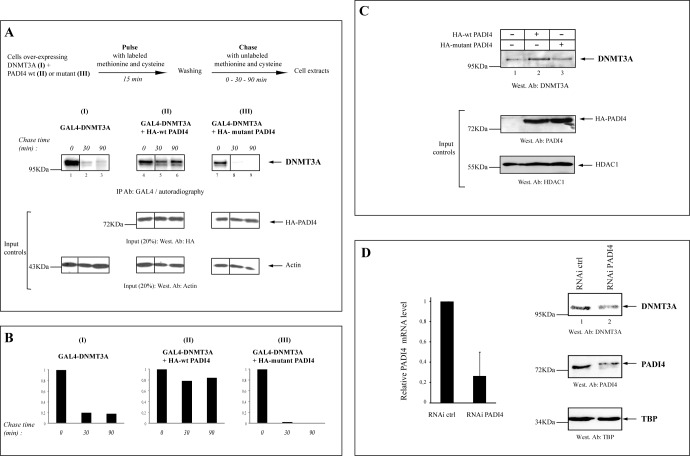
PADI4 stabilizes the DNMT3A protein. (A) Upper part, outline of the experimental procedure used to monitor DNMT3A stability. Pulse-chase with radioactive [^35^ S] methionine following transfection of 293T cells with either GAL4-DNMT3A alone ((I), lanes 1–3) or GAL4-DNMT3A together with wt HA-PADI4 ((II), lanes 4–6) or mutant HA-PADI4 ((II), lanes 7–9). Levels of the different proteins in the inputs were checked by western blotting with anti-HA (for PADI4 expression) or anti-actin antibody (Input controls). Actin was detected as a loading control, while probing with anti-HA demonstrated equal expression of wt and mutant HA-PADI4. Vertical lines indicate juxtaposition of lanes non-adjacent within the same blot, exposed for the same time. (B) Quantification of protein bands corresponding to DNMT3A on western blots. (C) PADI4 regulates the endogenous DNMT3A protein level. U2OS cells were transiently transfected with the expression vector encoding the wt or catalytically inactive mutant HA-tagged full-length PADI4. Nuclear extracts were then prepared and the level of DNMT3A was estimated by western blot analysis. HDAC1 was used as a loading control. (D) Endogenous levels of DNMT3A protein is decreased in PADI4 knockdown. Left, Validation of the PADI4-targeting RNAi by quantitative RT-PCR. Each RT-qPCR was normalized with respect to the expression of housekeeping gene GAPDH, and the level of each mRNA in the presence of the control RNAi was set at 1. Error bars represent SD of three independent experiments. Right, western blot showing reduction of endogenous DNMT3A in the presence of RNAi targeting PADI4. Validation of the PADI4-targeting RNAi at protein level by western blotting. TBP levels in inputs were checked to confirm that equal amounts of extract were used.

As shown above, cells ectopically expressing both DNMT3A and wt PADI4 showed a higher level of DNMT3A protein than did cells expressing DNMT3A alone (Figure 1B, Input controls). To see if this might reflect a role of PADI4 activity in stabilizing the DNMT3A protein, we performed pulse-chase experiments on 293T cells transfected with GAL4-DNMT3A, alone or with wt or mutant HA-PADI4 (Figure 3A, see scheme of the experiment). After 30 min of chase, mock-transfected cells showed a significantly reduced amount of ^35^S-labeled GAL4-DNMT3A (Figure 3A, (I), lanes 1–3), whereas in cells ectopically expressing both PADI4 and DNMT3A (Figure 3A, (II), lanes 4–6), DNMT3A displayed a longer half-life (>90 min). In cells expressing both DNMT3A and the C645S mutant (Figure 3A, (III), lanes 7–9), DNMT3A displayed a half-life similar to that observed in cells transfected with DNMT3A alone. Figure 3B shows quantification of the intensities of the protein bands corresponding to DNMT3A (Figure 3B) obtained from transfections with DNMT3A and/or wt or mutant PADI4. Such pulse-chase experiments were repeated several times. Another example is depicted in Supplementary Figure S3: in mock-transfected cells (lanes 1–3) and in cells expressing DNMT3A and the PADI4 mutant (lanes 7–9), DNMT3A displayed a reduced half-life (although the effect was less pronounced than in cells expressing DNMT3A alone, lane 2). In contrast, and as shown in Figure 3A, DNMT3A displayed a longer half-life in cells expressing wt PADI4 (Supplementary Figure S3, lanes 4–6).

These results suggest that wt PADI4 activity stabilizes DNMT3A. To further substantiate this, cells were transfected with a vector expressing HA-tagged wt or mutant PADI4, and nuclear extracts of the transfected cells were western blotted and probed for endogenous human DNMT3A. When overexpressed in these cells, wt PADI4 but not the mutant PADI4 was found to increase the level of endogenous DNMT3A (Figure 3C). In these experiments, HDAC1 was detected as a loading control and blots were probed with anti-HA to show the inputs of wt and mutant HA-PADI4 (Figure 3C, Input controls).

Finally, we sought to validate the above observations in untransfected cells. To this end, we evaluated DNMT3A protein levels in U2OS cells depleted of PADI4 by RNAi. Figure 3D shows efficient knockdown of PADI4, at both the mRNA and protein levels, as assessed by RT-qPCR and western blotting, respectively. As depicted in Figure 3D (right part), an RNAi targeting PADI4 caused the amount of endogenous DNMT3A protein to decrease, as compared to a control RNAi.

Together, our results indicate that PADI4 stabilizes the DNMT3A protein and that its catalytic activity is required to mediate this effect.

### PADI4 citrullinates DNMT3A in a region adjacent to the PWWP domain

**Figure 4.**
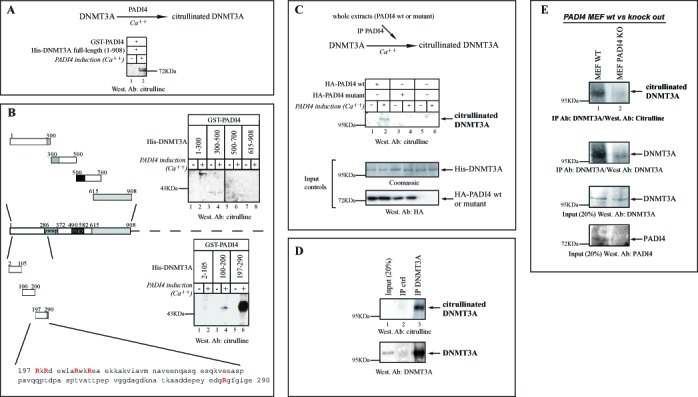
PADI4 citrullinates DNMT3A *in vitro* and *in vivo*, via a PWWP-adjacent region. (A) PADI4 fused to GST was used in *in vitro* deiminase activity assays to assess citrullination of His-DNMT3A full-length (aa 1–908) fusion protein used as substrate. The presence of citrullinated DNMT3A was revealed by western blotting with anti-modified citrulline antibody. (B) PADI4 citrullinates DNMT3A within an upstream PWWP region. *In vitro* deiminase assays were performed as in (A), this time using several rather large (200–300 aa) fragments. This revealed the N-terminal part of DNMT3A (amino acids 1–300) as the region citrullinated by PADI4 (upper panel). In the lower panel, smaller fragments within this 1–300 region were used. Schematic representation of the various His-fusions of DNMT3A used, shown with zooming-in on the identified citrullinated sequence 197–290, which contains 5 arginines. The vertical line indicates juxtaposition of lanes non-adjacent within the same blot, exposed for the same time. (C) Upper part, outline of the experimental procedure used to monitor citrullination of DNMT3A. U2OS cells were transiently transfected with the expression vector for wt or mutant HA-tagged PADI4. Nuclear extracts were prepared and tested for *in vitro* deiminase activity, using recombinant His-DNMT3A as a substrate. Coomassie staining was used as loading control. (D) Citrullination of endogenous human DNMT3A by PADI4 *in vivo*. As described in Figure 1C, lysates of untransfected U2OS cells were immunoprecipitated with anti-DNMT3A or control (IgG) antibodies. Endogenous and modified DNMT3A (citrullinated DNMT3A) were this time revealed, respectively, by western blotting with anti-DNMT3A and anti-modified citrulline antibodies. % of Input (20%) is shown. (E) Decreased citrullination and reduced expression of endogenous DNMT3A in PADI4-knockout MEFs. Nuclear lysates of wt and PADI4-knockout MEFs were immunoprecipitated with anti-DNMT3A. Endogenous and modified DNMT3A (citrullinated DNMT3A) were revealed by western blotting with anti-DNMT3A and anti-citrulline antibodies, respectively. PADI4 expression in wt versus knockout MEFs is shown after western blotting with anti-PADI4.

As the catalytic activity of PADI4 appears necessary to promote DNMT3A stabilization, we next examined whether PADI4 can directly citrullinate DNMT3A. Experiments were performed with either recombinant or endogenous DNMT3A protein as substrate (Figure 4). First, after incubation of recombinant full length His-DNMT3A (His-DNMT3A fl) with GST-PADI4, citrullinated DNMT3A was detected by western blotting (see Figure 4A, upper part for scheme of the experiment). In the presence of calcium ions, PADI4 was found to citrullinate His-DNMT3A fl *in vitro* (Figure 4A).

Next, we sought to narrow down the DNMT3A region citrullinated by PADI4. For this, we first used rather large fragments (200–300 aa) to scan DNMT3A in PADI4-mediated citrullination assays. As shown in Figure 4B (upper part, lane 2), we identified the N-terminal portion of DNMT3A (amino acids 1–300) as the region citrullinated by PADI4. No signal was detected without Ca++, necessary for PADI4 catalytic activity (Figure 4B, upper part, lanes 1,3,5,7,9) or with other fragments (Figure 4B, upper part, lanes 4,6,8). In a second step, we used smaller fragments within this 1–300 region (see scheme in Figure 4B). This enabled us to identify the stretch spanning 197–290, which contains 5 arginines, as the main citrullinated fragment of DNMT3A (Figure 4B, lower part).

To evaluate the citrullinated level of DNMT3A, we performed *in vitro* citrullination assays to compare the action of recombinant GST-fused PADI4 on DNMT3A and histone H3, whose citrullination is well described (15). Modified DNMT3A and H3 were detected by western blotting with anti-modified citrulline antibody. As presented in Supplementary Figure S4, we found modification of DNMT3A by PADI4 to be significant as compared to H3. These results, we feel, are well in line with our overall data on citrullination of DNMT3A by PADI4.

To further substantiate the citrullination of DNMT3A, cells were transfected with a plasmid expressing wt or mutant PADI4. Whole-cell extracts were prepared from the transfected cells and the wt or mutant PADI4 was immunoprecipitated. His-DNMT3A protein was added to the immunoprecipitate and tested for citrullination (Figure 4C, scheme of the experiment). Citrullination of DNMT3A was detected in the presence of wt PADI4 (Figure 4C, lane 2) but not in the presence of mutant PADI4 or in the absence of any PADI4 variant (Figure 4C, lanes 3–6). The Coomassie-blue-stained gel showed that recombinant DNMT3A protein was present in equal amount in all reactions (Figure 4C, Input controls). Overexpression of wt and mutant PADI4 was checked by western blotting with anti-HA antibody (Figure 4C, Input controls).

Having shown endogenous PADI4-DNMT3A association from untransfected U2OS cells (cf. Figure 1C), we next examined whether endogenous DNMT3A is citrullinated in this same cellular system. For this, as performed before (see Figure 1C), DNMT3A protein was immunoprecipitated from untransfected U2OS cells and subjected to western blotting, but this time with anti-citrulline antibody (Figure 4C). In this experiment on U2OS cells, we first had to immunoprecipitate DNMT3A to be able to detect high levels of citrullinated DNMT3A. Detection of citrullinated proteins on western blots can vary according to the citrullinated protein of interest and/or the cel line used (this has been observed, for exemple, for histone H3 in MCF7 cells (15); see also Figure 4E below with MEFs).

Lastly, using MEFs derived from PADI4-deficient mice (35), we wished to further investigate PADI4-mediated DNMT3A citrullination and the impact of PADI4 on the DNMT3A protein level. The following experiments confirmed both PADI4-mediated citrullination and increased expression of DNMT3A (Figure 4E): (i) Citrullination of DNMT3A was found to be strongly decreased in PADI4-knockout vs wt MEFs; (ii) protein expression of DNMT3A was severely compromised in PADI4-knockout. These data thus extend to a physiologically relevant system and confirm our results depicted in Figure 4A-D.

### PADI4 upregulates DNA methylation

**Figure 5.**
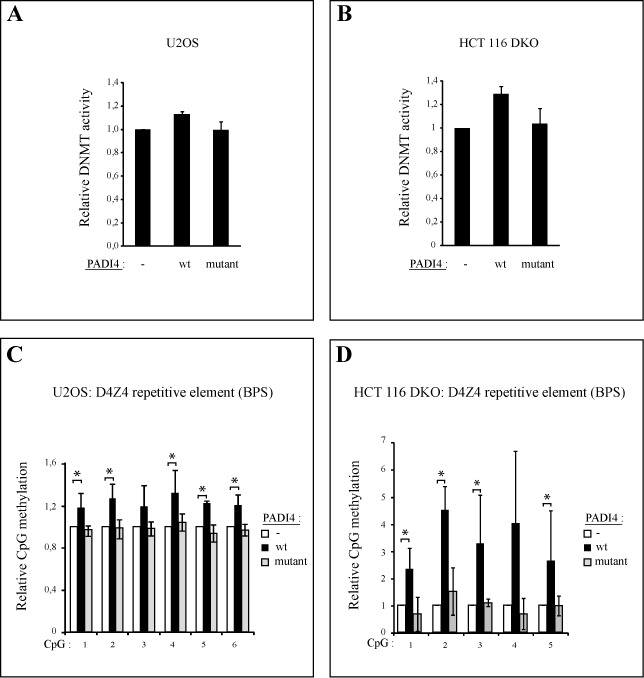
PADI4 increases DNMT activity and DNA methylation at the D4Z4 repeat, while a catalytically inactive mutant PADI4 does not. (A) PADI4 increases DNMT activity (DNMT). U2OS (A) and HCT 116 DKO cells (B) were stably transfected with the pLPC/control vector (-), the pLPC/PADI4 vector (wt) or the pLPC/PADI4 mutant vector (mutant). *In vitro* DNMT activity assays were performed. Experiments were done in triplicate. (C) PADI4, in contrast to its catalytically inactive mutant, increases DNA methylation at the D4Z4 repeat. Genomic DNA was prepared from U2OS (C) and HCT 116 DKO cells (D) harboring a control vector (-) or expressing wt PADI4 (wt) or mutant PADI4 (mutant). Values are means of three independent experiments. The methylation level of each control was set at 1 (see also raw pyrograms of representative experiments in Supplementary Figure S6A and B). Errors bars represent standard deviations of three independent biological experiments.

On the basis of the above data showing that PADI4 stabilizes the DNMT3A protein (Figure 3A) and increases its level (Figure 3C), we hypothesized that PADI4 overexpression might increase DNMT activity and thus DNA methylation. To test this hypothesis, U2OS cells were stably transfected with a vector expressing wt or mutant PADI4 (see Supplementary Figure S5A for western blotting controls). Nuclear extracts were prepared and assayed for *in vitro* DNA methylation activity with a CpG-rich DNA substrate (see Materials and Methods for details). As shown in Figure 5A, the global DNMT activity was higher in extracts of PADI4-overexpressing cells than in extracts of mock-transfected cells. When the catalytically inactive PADI4 mutant was overexpressed in cells instead of wt PADI4, the global DNMT activity was similar to that of mock-transfected cells (Figure 5A). It is worth noting that western blotting controls for the PADI4 overexpression level (cf. Supplementary Figure S5A) showed higher expression of the PADI4 mutant than of the wt, yet the PADI4 mutant failed to show increased DNMT activity as seen with the wt PADI4. This further shows the contribution of citrullination to the observed impact of PADI4 on global DNMT activity.

To observe the consequences of PADI4-mediated DNMT3A upregulation specifically, we used the colorectal cancer cell line HCT-116, where both the DNMT1 and DNMT3B DNMTs have been genetically disrupted (DKO cells) and where DNMT activity is minimal (38). Western blotting controls for PADI4 overexpression are shown in Supplementary Figure S5B. As depicted in Figure 5B, overexpression of wt PADI4 in these cells caused the DNMT activity, essentially mediated by DNMT3A, to increase. No such effect was observed in cells overexpressing the mutant PADI4 (Figure 5B). This suggests that PADI4 deiminase activity is required to upregulate DNMT activity.

Having established that PADI4 increases DNMT activity, we examined whether PADI4 might control the methylation of specific genomic sequences. First, we focused on the D4Z4 subtelomeric repeats, known to be regulated by methylation in cancer cells (39). We stably transfected U2OS cells with a vector expressing wt PADI4 or the catalytically inactive mutant and determined the methylation level of the D4Z4 macrosatellite repeat by bisulfite pyrosequencing. When wt PADI4 was overexpressed, a moderate but significant increase in D4Z4 methylation was observed (Figure 5C and Supplementary Figure S6A for primary pyrograms). When the same experiment was carried out on HCT 116 DKO cells, wt PADI4 overexpression caused methylation of the D4Z4 repeat to increase more substantially than in U2OS cells, whereas catalytically inactive PADI4 had no effect (Figure 5D and Supplementary Figure S6B for primary pyrograms). Provided that it is catalytically active, PADI4 thus increases the DNA methylation level of the D4Z4 repeat.

**Figure 6.**
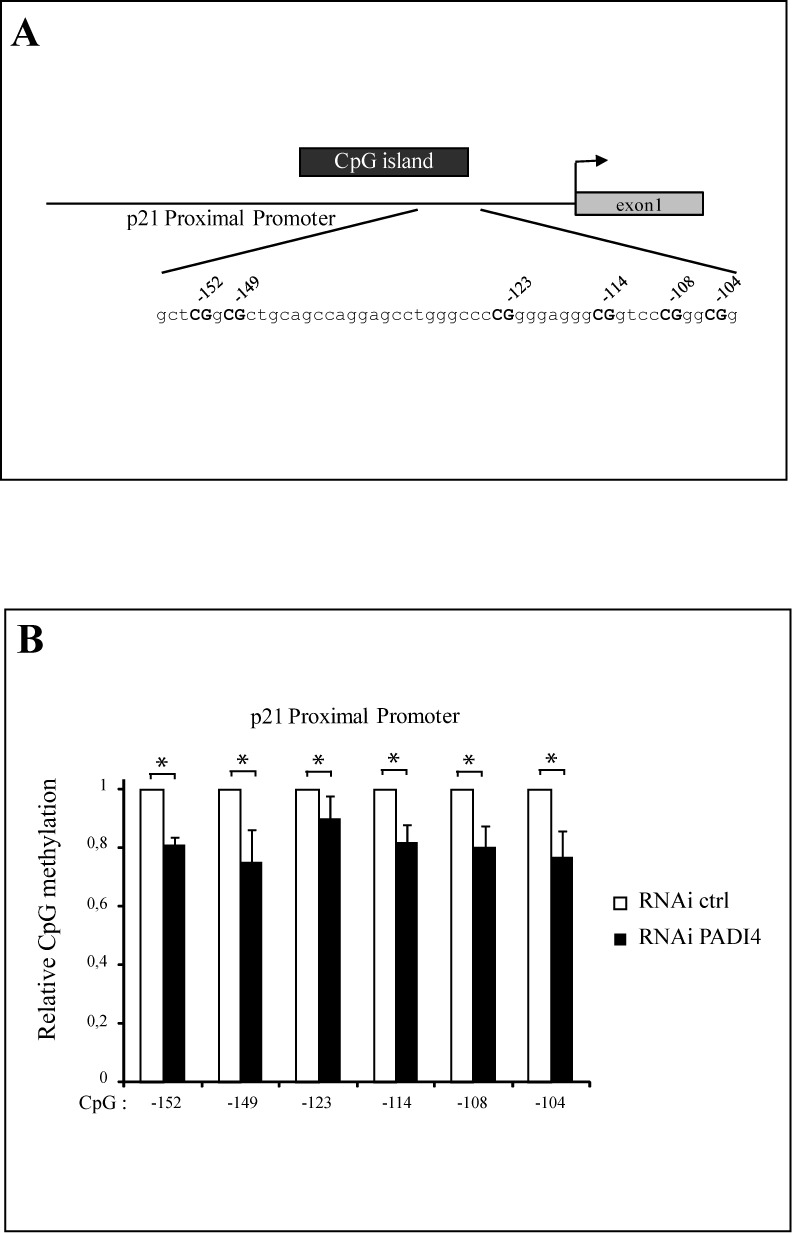
PADI4 influences methylation of the p21 promoter. (A) U2OS cells were stably transfected with the pRS/control vector (RNAi control) or the pRS/PADI4 vector (RNAi PADI4). After selection, the cells were harvested and quantitative RT-PCRs were performed. PADI4 transcript levels were normalized with respect to GAPDH and then divided by the normalized level recorded for control cells. Experiments were done in triplicate. (B) Genomic DNA was prepared from U2OS cells expressing control RNAi or PADI4 RNAi. Methylation analysis of the p21 promoter was then assessed by bisulfite pyrosequencing. Values are means of three independent experiments. Methylation level of each control was set to a value of 1 (primary pyrograms of representative experiments are shown in Supplementary Figure S7). Errors bars represent standard deviations of three independent biological experiments.

We next examined how PADI4 might control methylation of a non-repeat sequence. We focused on the p21 promoter, chosen because it is one of the promoter genes at which PADI4 has been shown to mediate citrullination of histone H3 (21,23). In addition, DNMT3A is known to be recruited to the p21 proximal promoter and methylate its promoter (10). To evaluate whether PADI4 might be involved in regulating p21 promoter methylation, we assessed the methylation status of the p21 proximal promoter in U2OS cells treated with an RNAi against PADI4 (cf. Figure 3D). We then analyzed by bisulfite pyrosequencing the methylation status of the p21 proximal promoter in a region known to be targeted by DNMT3A (Figure 6A and (10)). In this experiment (Figure 6B), PADI4 depletion led to a moderate but significant decrease in p21 promoter methylation (see also Supplementary Figure S7 for raw pyrogram data). Taken together, our data indicate that PADI4 upregulates DNA methylation in specific genomic regions.

## DISCUSSION

Our understanding of how specific genomic regions are targeted for *de novo* DNA methylation and how *de novo* DNMTs are regulated is still poor. It is becoming clear that other mechanisms besides the proposed recruitment of DNMTs by transcription factors and chromatin-based mechanisms (7) are involved in regulating DNMT activity. Previous studies have notably highlighted the possible influence of covalent PTMs (acetylation, SUMOylation, methylation, phosphorylation) on the function of DNMT1, although little is known about this influence (7,11). To date, no PTM except SUMOylation of DNMT3A and DNMT3B (14) is reported to target DNMT3A-family enzymes, and the reported findings were based only on *in vitro* assays. Here, on the basis of both *in vitro* and *in vivo* data, we identify citrullination as a novel DNMT3A-targeting PTM.

We report a close connection between PADI4 and DNMT3A, showing that DNMT3A interacts with PADI4 *in vitro* and *in vivo* and associates with PADI4-meditated histone deiminase activity. We further show that native PADI4 can deiminate (citrullinate) endogenous DNMT3A. This is found both in U2OS cells depleted of PADI4 and in a physiologically relevant model system, namely, MEFs derived from PADI4-deficient mice (35). A question that remains is how does PADI4-mediated citrullination affect on DNMT3A function? By eliminating the positive charge of the arginine side chain, the citrullination reaction may influence protein structure and function (40), notably altering the interactions of the modified protein with its partners (28,29). For instance, PADI4-catalyzed citrullination of the inhibitor of growth 4 (ING4) was recently shown to inhibit ING4-mediated activation of p53 by disrupting the interaction between ING4 and p53 (29). Conversely, citrullination can promote interaction of the target protein with neighboring proteins (28). It would thus be interesting to investigate whether citrullination of DNMT3A modulates its ability to interact with one or more of its well-known partners, such as HDAC1 (31), SUV39H1 (41) or Myc (10), involved in targeting DNMT3A and DNA methylation to specific regions of the genome. Further regarding the mechanisms by which PADI4-mediated citrullination might affect DNMT3A function, it is of interest that we have identified the region of DNMT3A lying just upstream from the PWWP domain as the primary citrullinated region. This region, spanning amino acids 197 through 290, contains 5 arginines, which might all potentially be citrullinated. It might be worth examining whether citrullination of residues in the 190–297 region affects known functions of the DNMT3A PWWP domain, such as its chromatin targeting function (42,43), its oligomerization (44) and its interactions with the H3 tail (45). It might also be worth assessing whether PADI4 influences DNMT3A localization, as shown in the reported case of one of its interactors, NPM1 (26). More precisely, it has been shown that citrullination of NPM1 by PADI4 results in NPM1 translocation from the nucleoli to the nucleoplasm (26). DNMT3A is highly localized to a subset of nucleosomes containing methylated CpG sites (46) and it could be interesting to assess whether citrullination of DNMT3A by PADI4 can affect its nucleosomal localization. This could be done by means of immunocytochemistry as well as by native ChAP assays performed on nucleosomes isolated from MNase-digested nuclei followed by direct sequencing of the immunoprecipitated DNA, or by NOMe-seq to provide precise DNA methylation and nucleosome positioning information.

Most interestingly, we find that PADI4 overexpression leads to an increased level of DNMT3A protein, whether synthesis of the latter is due to expression of an endogenous or a transduced gene. Notably, this is observed not only in U2OS cells depleted of PADI4 but also PADI4-knockout MEFs. (We wish to point out that we also observed an increased PADI4 protein level in cells overexpressing DNMT3A, suggesting a positive feedback loop between PADI4 and DNMT3A. While the present work focuses on PADI4-mediated regulation of DNMT3A, additional studies might be worth pursuing to investigate the potential modulation of PADI4 by DNMT3A.) Our pulse-chase data show that in the presence of overexpressed PADI4, the half-life of the DNMT3A protein is increased. As a catalytically defective PADI4 mutant fails to produce such effects and as we show that PADI4 can directly citrullinate DNMT3A, we suggest that citrullination of DNMT3A by PADI4 increases the stability of DNMT3A, perhaps by modulating its susceptibility to degradation as shown for other proteins known to undergo citrullination (29,47). In principle, however, it remains possible that PADI4-mediated citrullination might act indirectly on DNMT3A, and thereby on DNMT activity. To date, very little is known regarding how PTMs might affect DNMT stability. All we know so far concerns DNMT1 (7), whose methylation at Lys 147 by SET7 and phosphorylation at Ser 143 by Akt kinase have been shown to regulate its stability and degradation, thus affecting DNA methylation (12).

We have also investigated the functional consequences of PADI4-mediated DNMT3A upregulation. We show that when stably transfected with an expression vector encoding PADI4, U2OS cells show enhanced global DNMT activity and that HCT116 DKO cells (lacking DNMT1 and DNMT3B) show increased DNMT3A activity, the effect being moderate but significant in both cases. The results of our bisulfite pyrosequencing experiments further show that PADI4 increases methylation of the D4Z4 repeat, whereas PADI4 depletion somewhat decreases p21 promoter methylation, the effect being moderate but significant. PADI4 is reported to be recruited to the p21 promoter for citrullination of histones (21,23). Our results reveal that PADI4 can also citrullinate DNMT3A, increasing its stability and that this correlates with higher DNMT-mediated methylation of the p21 promoter. DNMT3A is thus a new example of a non-histone protein targeted by PADI4. Our data on the p21 promoter suggest that PADI4 is required to maintain DNA methylation at this locus. DNMT3A is often regarded solely as a *de novo* methyltransferas, but several works indicate that this classification is too simplistic. For example, DNMT3A is also clearly involved in maintaining DNA methylation (38,42,46). One should also consider the possibility that the effect of PADI4 knockdown on DNA methylation of the p21 promoter might be indirect. For example, it might be mediated via the well-known citrullination of histones by PADI4, which can lead to gene silencing (15). Our results might have important consequences for human disease, particularly cancer, as PADI4 can be upregulated or downregulated in various tumors (48–50). It is well known that cancer cells are characterized by both hypermethylation of specific promoters and global hypomethylation (51). Our discovery that higher PADI4 levels lead to increased DNMT3A expression and stability might explain, at least partially, the observed hypermethylation of some promoters in cancers. Conversely, decreased PADI4 expression has recently been associated with global hypomethylation in hepatocellular carcinoma during hepatitis B virus exposure (50). Future work will be needed to address this exciting potential role of PADI4 deregulation in causing DNA methylation alterations in cancers.

In conclusion, our results highlight an additional non-histone protein that PADI4 is able to citrullinate. Our findings also reveal a new mode of regulation of DNMT3A: protein citrullination. They thus shed new light on how PTMs might influence the function of *de novo* DNMTs.

## SUPPLEMENTARY DATA

Supplementary Data are available at NAR Online.

SUPPLEMENTARY DATA
