# Supplementary material for: Citrullination of DNMT3A by PADI4 regulates its stability and controls DNA methylation
Source: Nucleic Acids Res. 2014 Jun 21;42(13):8285–96. doi: 10.1093/nar/gku522 (PMC4117755; doi:10.1093/nar/gku522)
Supplement: SUPPLEMENTARY DATA [file supp_42_13_8285__index.html]

Citrullination of DNMT3A by PADI4 regulates its stability and controls DNA methylation — SUPPLEMENTARY DATA 

# Citrullination of DNMT3A by PADI4 regulates its stability and controls DNA methylation

## SUPPLEMENTARY DATA

**Files in this Data Supplement:**

- Supplementary Figure
- Supplementary Table
