# Supplementary material for: Citrullination of DNMT3A by PADI4 regulates its stability and controls DNA methylation
Source: Nucleic Acids Res. 2014 Jun 21;42(13):8285–96. doi: 10.1093/nar/gku522 (PMC4117755; doi:10.1093/nar/gku522)
Supplement: SUPPLEMENTARY DATA [file supp_gku522_nar-03653-v-2013-File013.pdf]

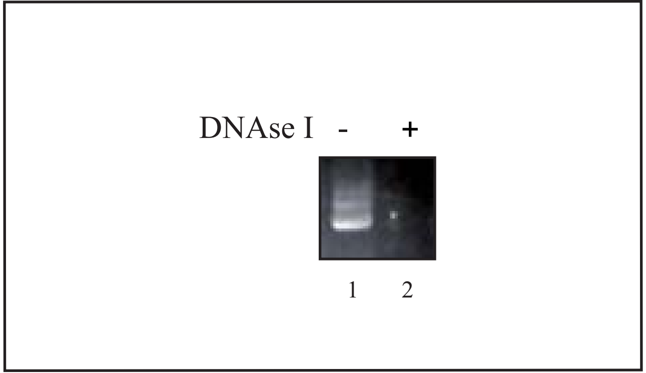

Supplementary Figure 1 (related to Figure 1C): Control of DNAse I efficacity. Plasmidic DNA was incubated with (lane 2) or without (lane 1) an excess of DNAse I for 1h at 37 C. This shows that endogenous PADI4-DNMT3A interaction is not due to indirect interactions mediated solely by DNA.

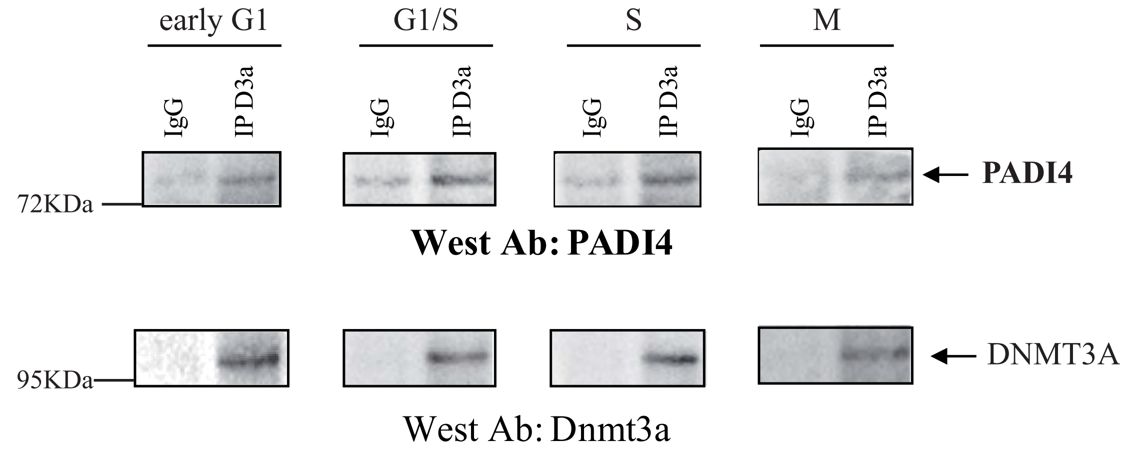

Supplementary Figure 2 (related to Figure 1): PADI4 co-immunoprecipitates with DNMT3A in various cell cycle phases. U2OS were treated with nocodazole or with thymidine to obtain cells in early G1, G1/S, S or in M phase. Cell extracts were immunoprecipitated with rabbit IgG or anti-DNMT3A and probed with antibodies against PADI4.

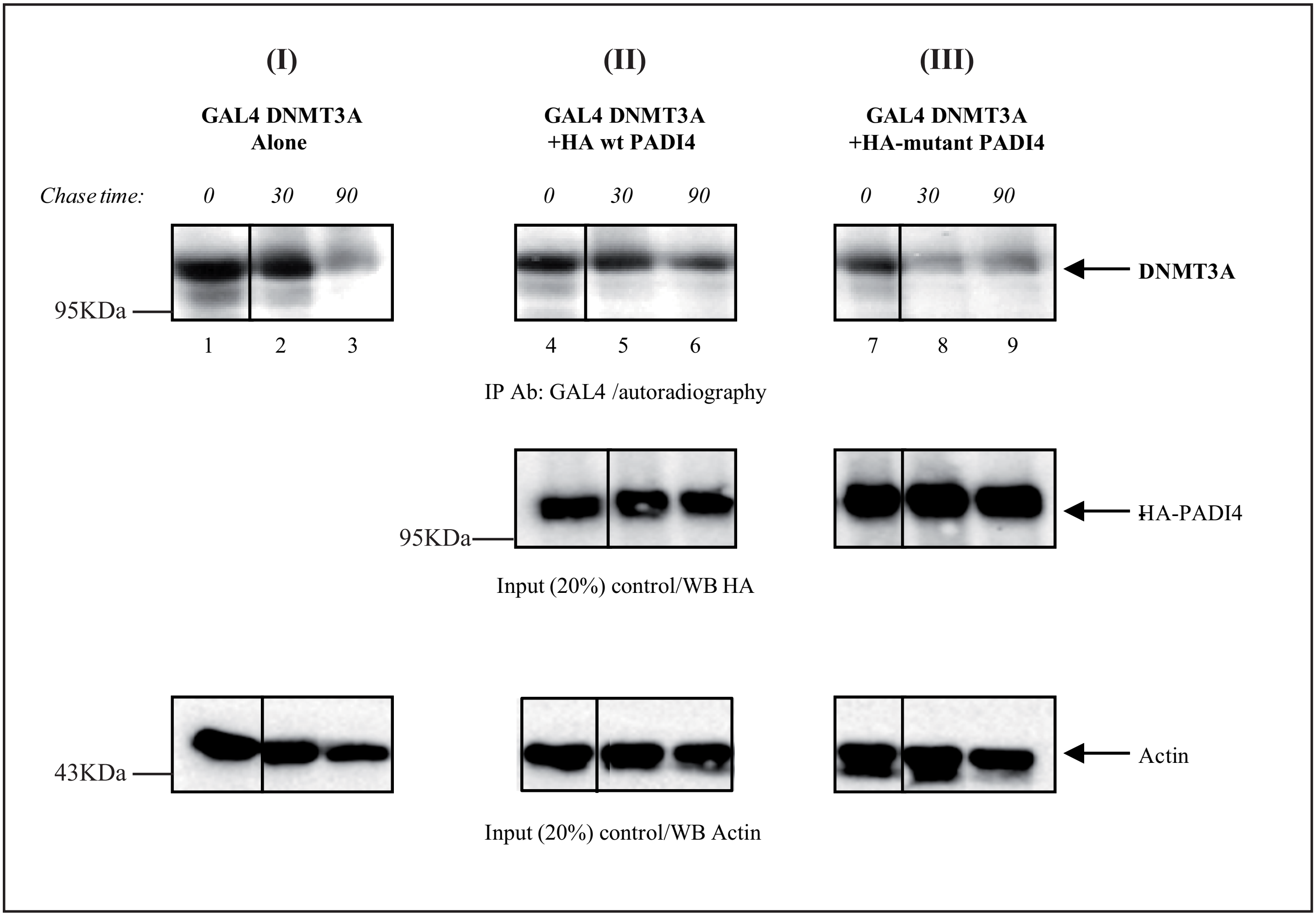

Supplementary Figure 3 (related to Figure 3A). Reproducibility of pulse-chase experiments. Pulse chase with radioactive methionine following transfection of 293T cells with either GAL4-DNMT3A alone ((I), lanes 1-3 or GAL4-DNMT3A together with wt HA-PADI4 ((II) lanes 4-6) or mutant HA-PADI4 ((III), lane 7-9). Levels of the different proteins in the inputs were checked by western blotting with anti-HA (for PADI4 expression) or anti-actin antibody (Input controls). Actin was detected as a loading control, while probing with anti-HA demonstrated equal expression of wt and mutant HA-PADI4. Vertical lines indicate juxtaposition of lanes non-adjacent within the same blot, exposed for the same time. These results show that, as observed before (cf. Fig. 3A), cells expressing PADI4 wt and DNMT3A displayed a longer DNMT3A half-life (lanes 4-6).

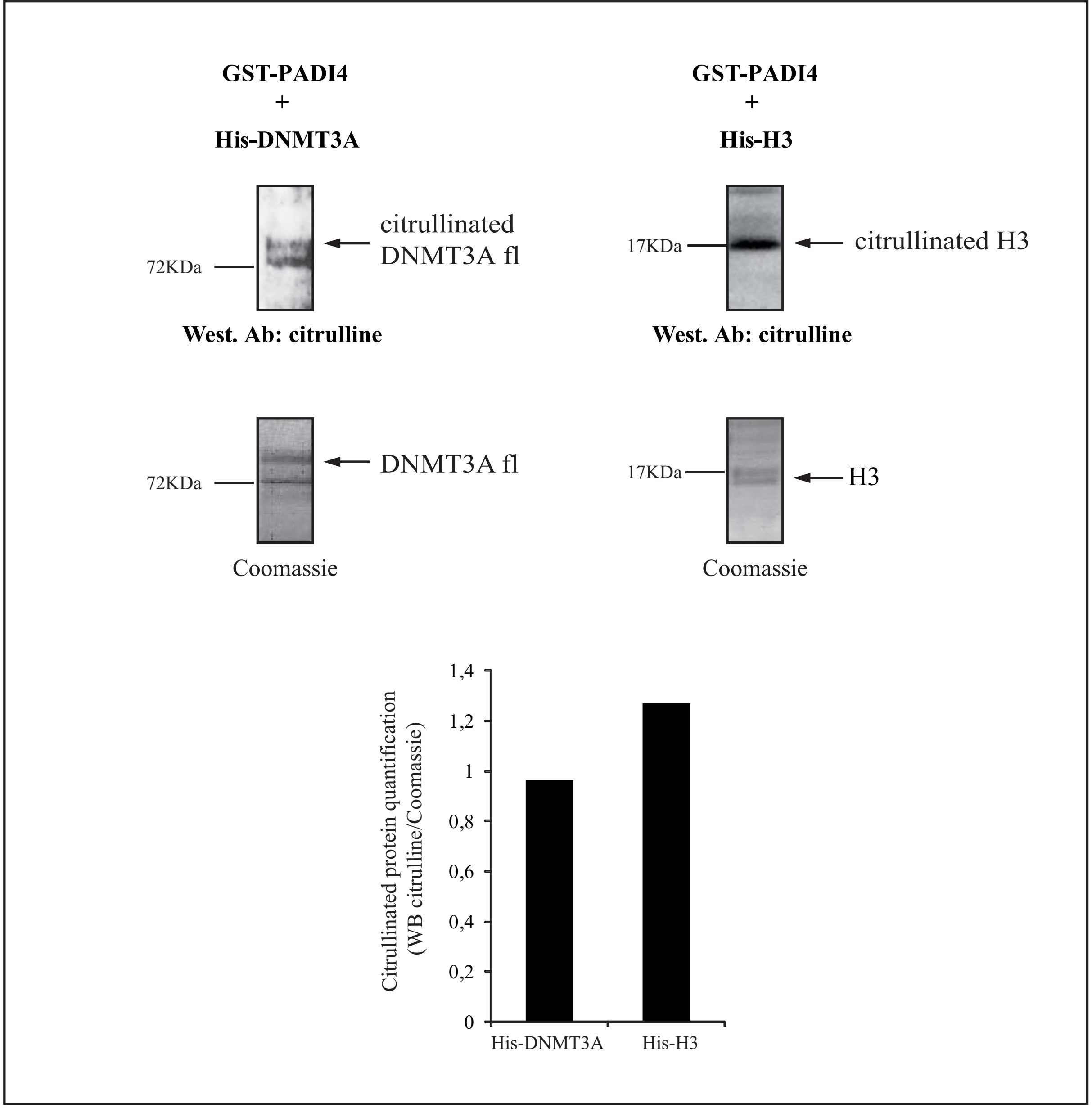

Supplementary Figure 4 (related to Figure 4A). Citrullination of DNMT3A is significant as compared to modification of histone H3by PADI4**.** Full-length PADI4 fused to GST was tested for *in vitro* deiminase activity in the presence of similar amounts of recombinant His-DNMT3A full-length (left part) or His-Histone H3 fusion protein (right part). The presence of citrullinated DNMT3A or Histone H3 was revealed by western blotting with anti-modified citrulline antibody. A Coomassie blue-stained gel shows fusion proteins. Quantification of citrullinated DNMT3A or H3 is shown below and was estimated as a ratio of the level of citrullination observed after western blotting with the anti-modified citrulline antibody to the loaded amount of His-tagged DNMT3A full-length or His-tagged H3.

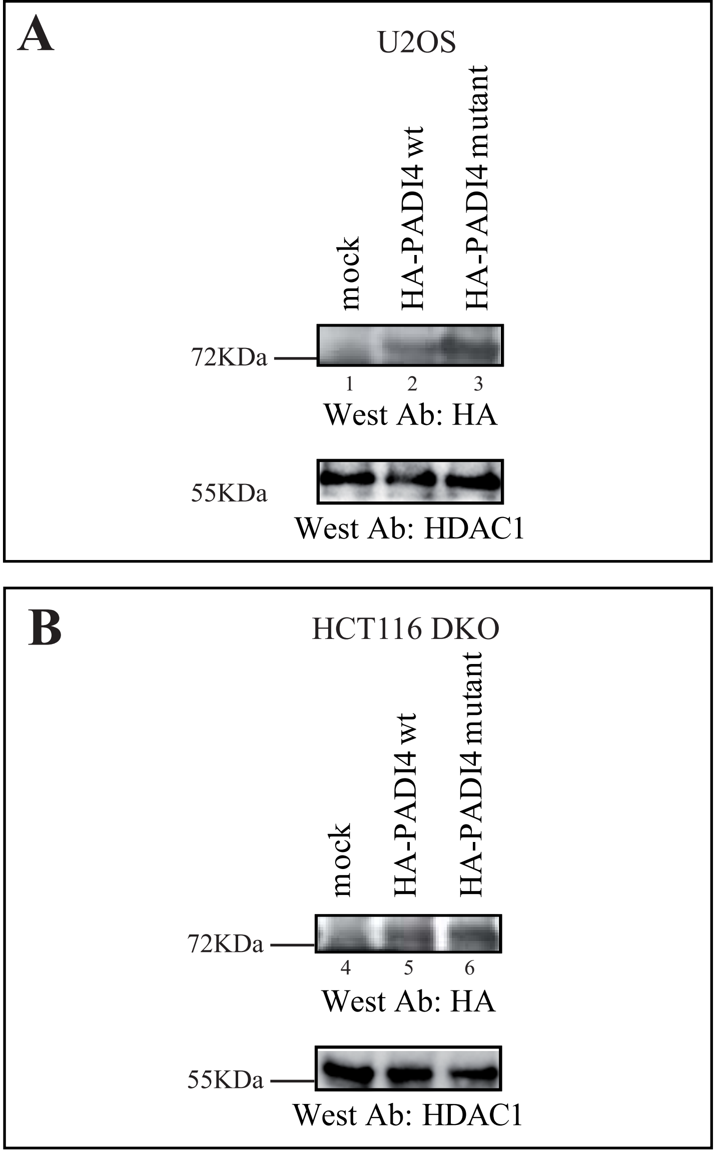

Supplementary Figure 5 (related to pyrosequencing shown in Figure 5A and B respectively). Control of overexpression of PADI4 wt and mutant in U2OS (A) and HCT116 DKO (B). Levels of the different proteins in the inputs were checked by western blotting with anti-HA (for PADI4 expression) or anti-HDAC1 antidody (Input controls). HDAC1 was detected as a loading control, while probing with anti-HA demonstrated equal expression of wt and mutant.

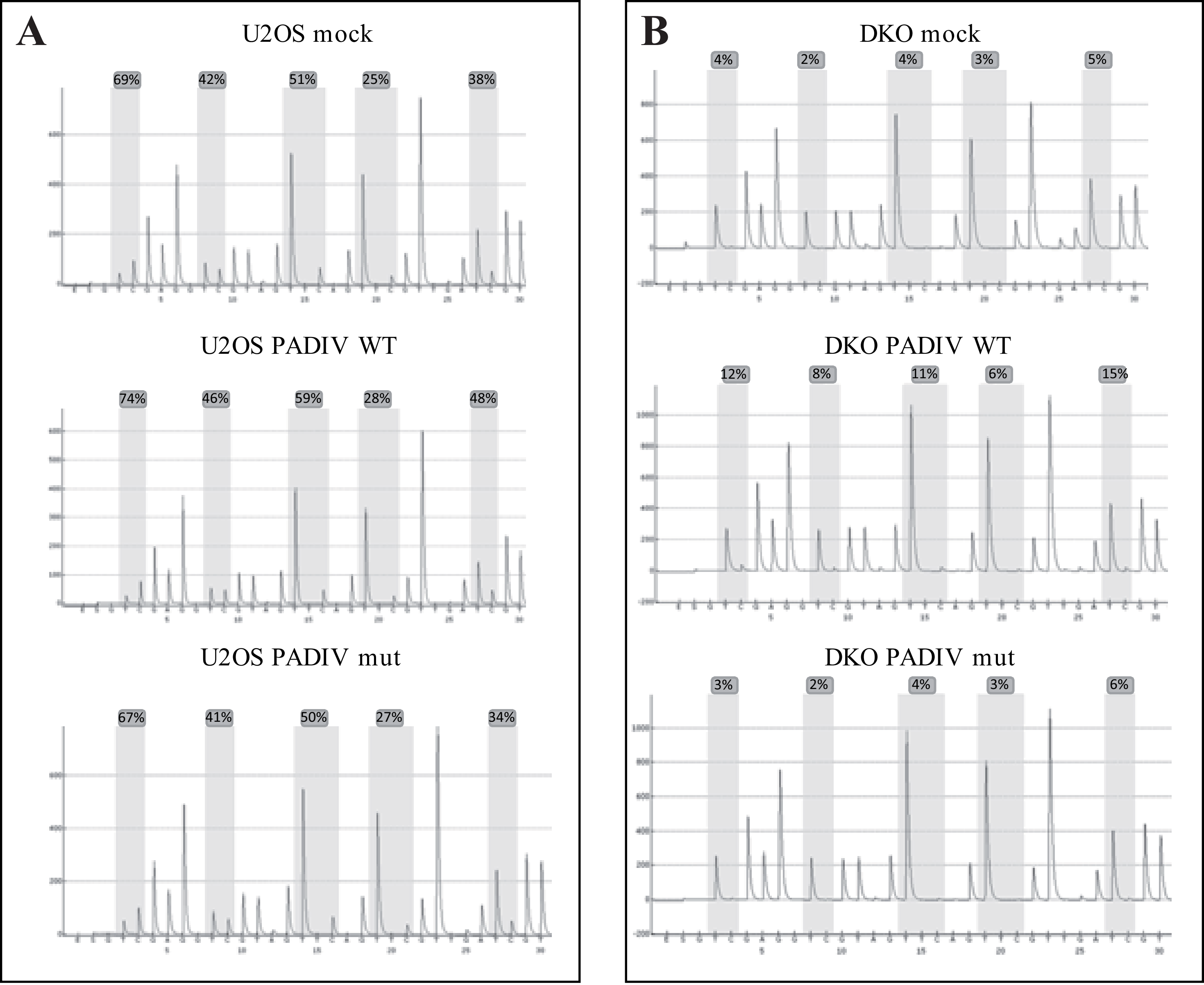

Supplementary Figure 6 (related to pyrosequencing shown in Figure 5A and B respectively). Control of overexpression of PADI4 wt and mutant in U2OS (A) and HCT116 DKO (B). Levels of the different proteins in the inputs were checked by western blotting with anti-HA (for PADI4 expression) or anti-HDAC1 antidody (Input controls). HDAC1 was detected as a loading control, while probing with anti-HA demonstrated equal expression of wt and mutant.

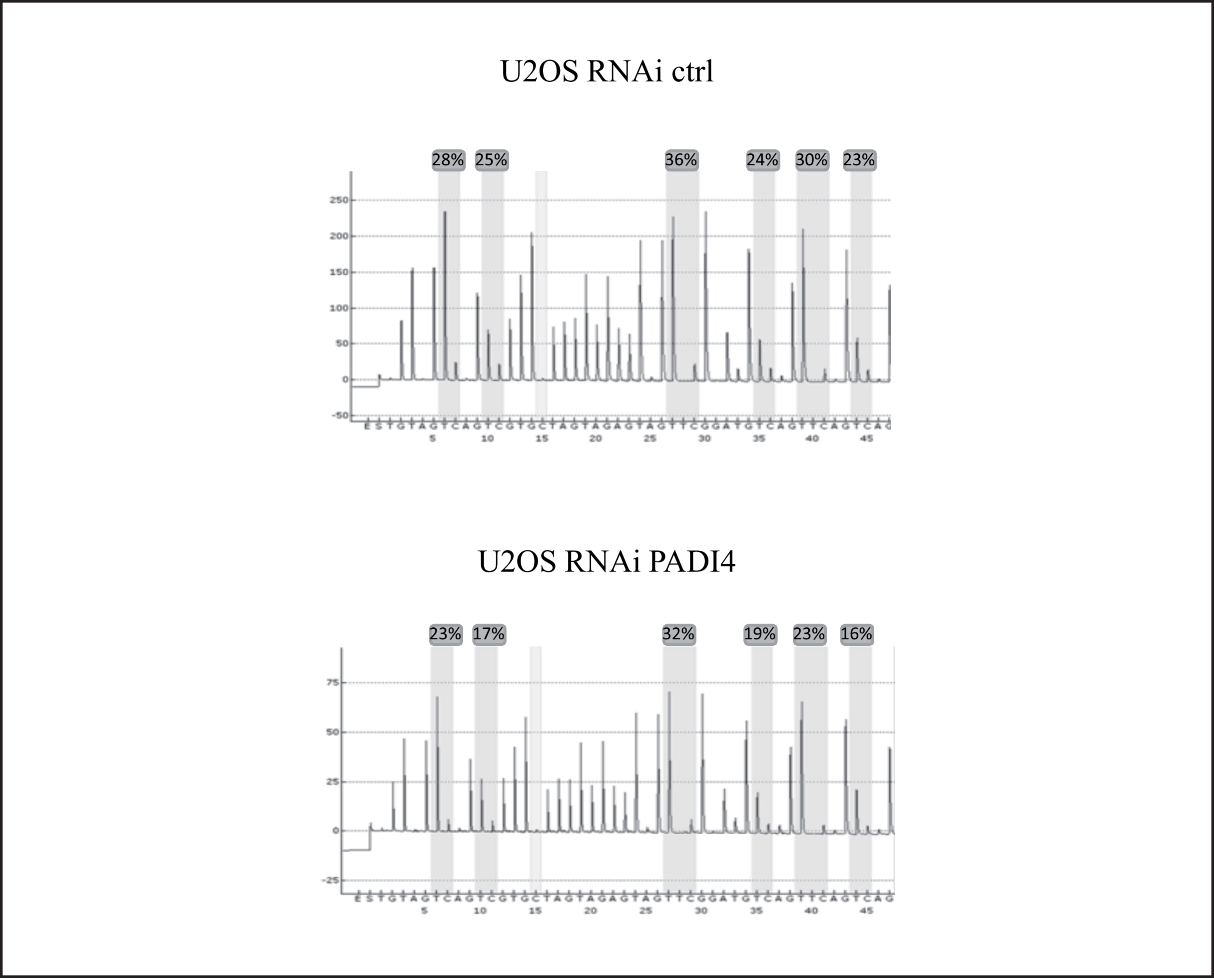

Supplementary Figure 7 (related to pyrosequencing shown in Figure 6). Representative pyrogramms of p21 promoter.
