# Supplementary material for: Citrullination of DNMT3A by PADI4 regulates its stability and controls DNA methylation
Source: Nucleic Acids Res. 2014 Jun 21;42(13):8285–96. doi: 10.1093/nar/gku522 (PMC4117755; doi:10.1093/nar/gku522)
Supplement: SUPPLEMENTARY DATA [file supp_gku522_nar-03653-v-2013-File012.doc]

## Supplementary Table 1: Primer lists and sequences.

| **Name** | **Sequence 5'-3'** | **Target** | **Application** |
| --- | --- | --- | --- |
| PADI4 R | gacaaagtgagggtgtttca | Human PADI4 | qPCR expression |
| PADI4 F | agaagtccatgttgtgcttt | Human PADI4 | qPCR expression |
| GAPDH F | TGCACCACCAACTGCTTAGC | Human GAPDH | qPCR expression |
| GAPDH R | GGCATGGACTGTGGTCATGAG | Human GAPDH | qPCR expression |
| shRNA PADI4 R | GATCCCCGCGAAGACCTGCAGGACATTTCAAGAGAATGTCCTGCAGGTCTTCGCTTTTTA | Human PADI4 | RNAi |
| shRNA PAD4 F | AGCTTAAAAAGCGAAGACCTGCAGGACATTCTCTTGAAATGTCCTGCAGGTCTTCGCGGG | Human PADI4 | RNAi |
| Pyr p21 F | AGGAGGGAAGTGTTTTTTTGTAGTA | Human p21 | Pyrosequencing |
| Pyr p21 R | [Btn]CCAACTCCCACTCCACAAAAAACTA | Human p21 | Pyrosequencing |
| Pyr p21 Seq | TGTTGGAATTAGGTTAGGTTTA | Human p21 | Pyrosequencing |
| Pyr D4Z4 R | GGTGGTTYGGGGTAGGG | Human D4Z4 | Pyrosequencing |
| Pyr D4Z4 F | [Btn]CCCAAAAAAAAATAACAATTCTC | Human D4Z4 | Pyrosequencing |
| Pyr D4Z4 Seq | GGGAATATTTGGTTGGTTA | Human D4Z4 | Pyrosequencing |
